# Supplementary material for: Comparative multiomics analysis of cell physiological state after culture in a basket bioreactor
Source: Sci Rep. 2022 Nov 23;12:20161. doi: 10.1038/s41598-022-24687-4 (PMC9686226; doi:10.1038/s41598-022-24687-4)
Supplement: Supplementary file 1 — Supplementary Information 1. [file 41598_2022_24687_MOESM1_ESM.zip › raw data/Transcriptomic raw data/6.Enrichment/1.GO/TE1vsTC1/GSEA/TC1/LIPID_CATABOLIC_PROCESS/LIPID_CATABOLIC_PROCESS(GO_0016042).html]

Details for gene set LIPID\_CATABOLIC\_PROCESS(GO:0016042)[GSEA]


|  || Dataset | readcount.sample |
| Phenotype | sample.cls |
| Upregulated in class | TC1 |
| GeneSet | LIPID\_CATABOLIC\_PROCESS(GO:0016042) |
| Enrichment Score (ES) | -0.24910474 |
| Normalized Enrichment Score (NES) | -1.0 |
| Nominal p-value | 0.0 |
| FDR q-value | 1.0 |
| FWER p-Value | 1.0 |
Table: GSEA Results Summary

  

Fig 1: Enrichment plot: LIPID\_CATABOLIC\_PROCESS(GO:0016042)
  
Profile of the Running ES Score & Positions of GeneSet Members on the Rank Ordered List

  

| PROBE | DESCRIPTION   (from dataset) | GENE SYMBOL | GENE\_TITLE | RANK IN GENE LIST | RANK METRIC SCORE | RUNNING ES | CORE ENRICHMENT || 1 | 103221411 | PLA2G7 |  |  | 321 | Infinity | -0.0134 | No |
| 2 | 103246657 | PLCB1 |  |  | 4679 | 1.438 | -0.0668 | No |
| 3 | 103243017 | ACOX1 |  |  | 4880 | 1.413 | 0.0629 | No |
| 4 | 103246100 | PLA2G4B |  |  | 11866 | 0.902 | -0.1606 | Yes |
| 5 | 103236105 | PLA2G12A |  |  | 11951 | 0.897 | -0.0764 | Yes |
| 6 | 103246430 | ACOX3 |  |  | 12134 | 0.885 | 0.0024 | Yes |
| 7 | 103233299 | PLCB3 |  |  | 12991 | 0.833 | 0.0459 | Yes |
| 8 | 103234777 | LIPE |  |  | 13663 | 0.791 | 0.0936 | Yes |
| 9 | 103225342 | PAFAH2 |  |  | 13921 | 0.774 | 0.1580 | Yes |
| 10 | 103229484 | GALC |  |  | 15396 | 0.683 | 0.1592 | Yes |
| 11 | 103227815 | ACOX2 |  |  | 16900 | 0.576 | 0.1486 | Yes |
| 12 | 103230418 | PLA2G4A |  |  | 18290 | 0.461 | 0.1318 | Yes |
| 13 | 103235528 | PLA2G4C |  |  | 19975 | 0.276 | 0.0837 | Yes |
| 14 | 103245842 | PLCB2 |  |  | 20144 | 0.251 | 0.1008 | Yes |
| 15 | 103245774 | PLA2G4F |  |  | 21401 | 0.000 | 0.0447 | Yes |
| 16 | 103245777 | PLA2G4E |  |  | 22010 | 0.000 | 0.0175 | Yes |
Table: GSEA details
[plain text format]

  

Fig 2: LIPID\_CATABOLIC\_PROCESS(GO:0016042)
  
Blue-Pink O' Gram in the Space of the Analyzed GeneSet

  

Fig 3: LIPID\_CATABOLIC\_PROCESS(GO:0016042): Random ES distribution
  
Gene set null distribution of ES for
 **LIPID\_CATABOLIC\_PROCESS(GO:0016042)**
